# Resveratrol delays postovulatory aging of mouse oocytes through activating mitophagy

**DOI:** 10.18632/aging.102551

**Published:** 2019-12-13

**Authors:** Jilong Zhou, Zhouyiyuan Xue, Hai-Nan He, Xin Liu, Shu-Yuan Yin, Dan-Ya Wu, Xia Zhang, Heide Schatten, Yi-Liang Miao

**Affiliations:** 1 Institute of Stem Cell and Regenerative Biology, College of Animal Science and Veterinary Medicine, Huazhong Agricultural University, Wuhan 430070, China; 2 Key Laboratory of Agricultural Animal Genetics, Breeding and Reproduction, Huazhong Agricultural University, Ministry of Education, Wuhan 430070, China; 3 Experimental Veterinary Medicine Education, Huazhong Agricultural University, Wuhan 430070, China; 4 The Cooperative Innovation Center for Sustainable Pig Production, Wuhan 430070, China; 5 Department of Veterinary Pathobiology, University of Missouri, Columbia, MO 65211, USA

**Keywords:** resveratrol, postovulatory aging, mitophagy, FoxO3a

## Abstract

Resveratrol (3,5,4′-trihydroxystilbene, RSV) is a natural potential anti-aging polyphenolic compound frequently used as a nutritional supplement against several diseases. However, the underlying mechanisms by which resveratrol regulates postovulatory aging of oocytes are still insufficiently known. In this study, we found that resveratrol could delay postovulatory aging and improve developmental competence of oocytes through activating selective mitophagy in the mouse. Resveratrol could maintain spindle morphology but it disturbed cortical granule (CG) distribution during oocyte aging. This might be due to upregulated mitophagy, since blocking mitophagy by cyclosporin A (CsA) treatment affected oocyte quality by damaging mitochondrial function and it decreased embryonic development. In addition, we also observed an involvement of FoxO3a in regulating mitophagy in aging oocytes following resveratrol treatment. Taken together, our results provide evidence that mitophagy induced by resveratrol is a potential mechanism to protect against postovulatory oocyte aging.

## INTRODUCTION

After ovulation, mature oocytes only have a short optimal time span for fertilization to take place. If not fertilized in time, these oocytes will undergo a time-dependent quality degradation process, which is called “postovulatory aging” [1, 2]. In assisted reproduction technologies (ART), oocytes are inevitably subjected to postovulatory aging, which leads to poor embryonic development after fertilization [3], increases abortion rates [4] and decreases offspring longevity [5]. Although quite significant technical progress has been made to improve ART technologies, poor oocyte quality is the key factor closely associated with ART failure. Therefore, it is highly important to understand the underlying mechanisms in the oocyte aging process.

Resveratrol (RSV), a polyphenolic antioxidant found in a variety of plants, has attracted the attention of various researchers for its life-span-extending effects in budding yeast. It has been well proved that RSV can help against cardiovascular disease [6], cancer [7] and age-related deterioration [8]; however, only few studies so far have focused on the beneficial effects of RSV on reproduction. Recently, a study focused on middle-aged mice showing that short-time injection of RSV could effectively ameliorate oxidative stress-induced fragmentation and death of oocyte aging in the oviduct [9], suggesting a beneficial role of RSV in the reproductive health of older females. Furthermore, supplementation of RSV could effectively promote *in vitro* maturation (IVM) and embryonic development in mice [10], human [11], pigs [12] and cows [13]. These data suggest a wide clinical application prospect of RSV in both human ART and agricultural animal embryo engineering. Recently, several lines of research have indicated that RSV is associated with an improved quality of oocyte [14], and further helps to increase the embryonic development rate [15]. However, evaluation of oocyte quality regulated by RSV has not been studied systematically and the molecular mechanisms have not yet been fully elucidated.

Abnormal distribution and function of mitochondria is closely related to aging and many of the age-related diseases [16]. Unlike somatic cells, the oocyte contains a large number of mitochondria to meet the demand of energy production during oocyte maturation and subsequent embryonic development. Mitochondrial selective autophagy, known as mitophagy, is a major process for cells to maintain normal mitochondria quality and quantity [17]. Recent studies reported that RSV remarkably reduced cadmium-induced ROS generation and mitochondrial injury through the Sirt1/FoxO3a pathway [18]. However, current evidence does not indicate a direct involvement of FoxO3a-mediated mitophagy regulation in postovulatory oocyte aging following RSV administration. In this study, we tested our hypothesis that resveratrol could delay postovulatory aging of oocytes through activating mitophagy. In addition, we identified FoxO3a as an important factor involved in RSV-mediated mitophagy during postovulatory oocyte aging.

## RESULTS

### RSV improves the developmental competence of aged oocytes

To assess the beneficial effect of RSV on oocyte aging, we added different concentrations (0, 2, 5, 10, 20, 40 μM) of RSV to culture medium for *in vitro* aging (8 h) to test whether RSV treatment could delay oocyte aging. Previous studies showed that oocyte aging was associated with an increased susceptibility to be activated [19]. We thus performed parthenogenetic activation and the results showed that 10 μM RSV treatment significantly decreased the activation rate compared to the control (59.0 ± 4.7%, n = 99 vs. 81.1 ± 5.5%, n = 105; *p* < 0.05) (Supplementary Figure 1). However, high concentrations of RSV (20 μM, 40 μM) led to embryonic developmental arrest at early cleavage stages and further caused decreased blastocyst rate (28.0% ± 3.8%, n = 90 vs. 34.1% ± 4.9%, n = 101; *p* > 0.05, 17.2% ± 2.9%, n = 93 vs. 34.1% ± 4.9%, n = 101; *p* > 0.05, Supplementary Figure 2), indicating a toxic effect at high concentrations of RSV. These results suggested that administration of RSV delayed oocyte aging at 10 μM RSV which was selected for subsequent research.

**Figure 1:**
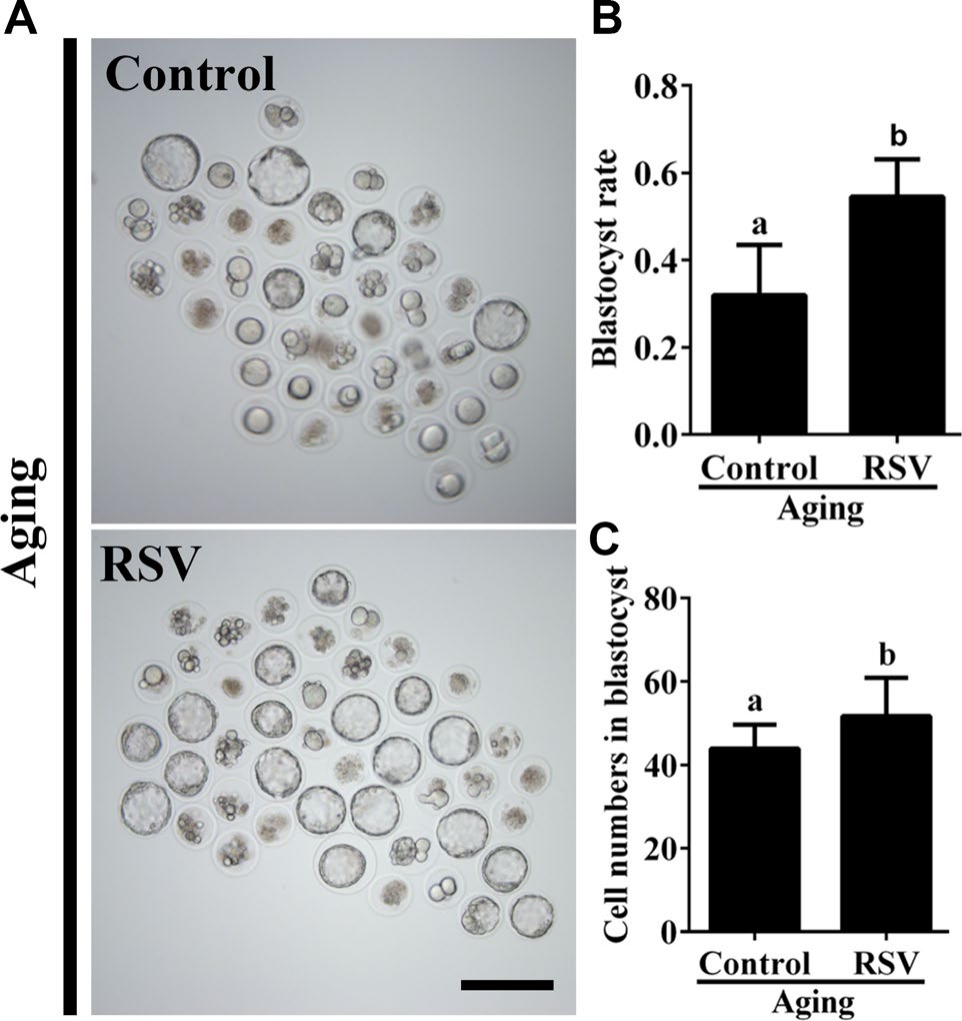
**RSV promotes the developmental competence of aged oocytes.** (**A**) Fresh oocytes were cultured *in vitro* with or without RSV for 8 h. These oocytes were parthenogenetic-activated and cultured for additional 3.5 days. Bar = 200 μm. (**B**) Statistical results of blastocyst rate in (**A**). (**C**) Quantitative analysis of blastocyst cell numbers in (**A**). Embryos were stained with DAPI. All experiments were performed in triplicates and the data represent the means ± SEM. * *p* < 0.05.

To further investigate the effect of RSV on developmental potential of *in vitro* cultured oocytes, we cultured the parthenogenetic-activated embryos for additional 84 h to assess the blastocyst formation. As shown in Figure 1A and 1B, RSV-treated oocytes displayed a higher blastocyst rate compared to the control (62.7 ± 4.1%, n = 105 vs. 35.9 ± 5.9%, n = 110; *p* < 0.05). In addition, we also found that the cell numbers of blastocysts after RSV treatment was significantly higher than that of the control (52.3 ± 2.7, n = 20 vs. 45.7 ± 2.6, n = 20; *p* < 0.05) (Figure 1C). Thus, our data demonstrated that RSV could delay postovulatory aging and improve the developmental competence in mouse oocytes.

### RSV maintains spindle morphology and affects CG distribution

**Figure 2:**
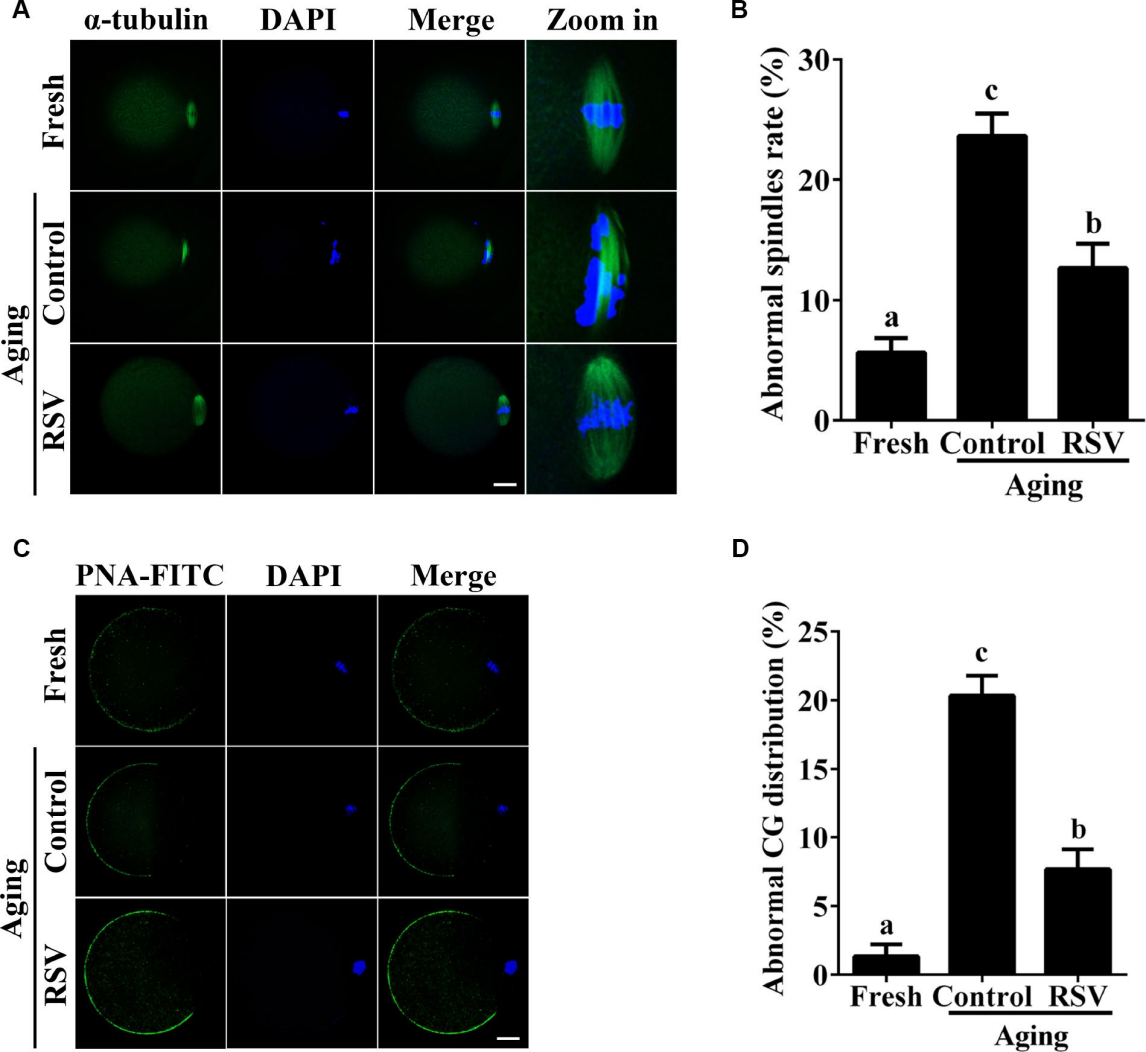
**RSV maintains the spindle morphology and affects the CG distribution.** (**A**) Spindles were imaged using anti-α-tubulin (green), and the chromosomes were counterstained with DAPI (blue). Bar = 200 μm. (**B**) Statistical analysis of abnormal spindle rate in (**A**). (**C**) CGs were imaged using anti-PNA (green), and the chromosomes were counterstained with DAPI. Bar = 200 μm. (**D**) Quantitative analysis of abnormal CG distribution in (**C**). Data are presented as means ± S.E.M of three independent experiments. Different lowercase letters represent the difference of expression levels that are significant (*P* < 0.05).

The highly dynamic spindle consisting of microtubules is critical for the normal alignment and separation of chromosomes [20]. Therefore, we tested the effect of RSV on spindle morphology during oocyte aging. As shown in Figures 2A and 2B, compared to fresh oocytes, the spindles of aged oocytes were disorganized or irregularly shaped with misaligned chromosomes (Abnormal rate: 23.7% ± 1.9, n = 97 vs. 5.7% ± 1.2, n = 103; *p* < 0.05), while spindles of oocytes in the RSV group appeared more compact and clear (Abnormal rate: 12.7 ± 2.0, n = 103 vs. 23.7% ± 1.9, n = 97; *p* < 0.05). Thus, RSV treatment affected spindle morphology during postovulatory oocyte aging by maintaining spindle integrity.

When oocytes were fertilized by sperm, CG contents are released into the perivitelline space (PVS) and CGs become combined with the zona pellucida (ZP), thereby avoiding polyspermic fertilization. Thus, we examined the CGs distribution during oocyte aging after RSV treatment. The results in Figure 2C and 2D show that CGs densely populated the area just beneath the oolemma, with typical CGs-free domains in fresh activated oocytes, while the aged oocytes showed a smaller CG distribution area compared to that of fresh oocytes (Abnormal rate: 20.3% ± 1.5, n = 83 vs. 1.3% ± 0.9, n = 91; *p* < 0.05). As expected, the decreased CGs distribution region was restored in oocytes upon RSV administration (Abnormal rate: 7.7% ± 1.5, n=102 vs. 20.3% ± 1.5, n = 83; *p* < 0.05). Since ZP hardening is one of the reasons for fertilization failure in aging oocytes [21], we investigated the effect of RSV on the ZP by the ZP hardening assay. Our results showed that the half-time (T50) for chymotrypsin-mediated ZP dissolution increased in aged oocytes compared to the control; however, it did not change significantly under RSV treatment (Supplementary Figure 2). Together, our results demonstrated that RSV treatment could maintain a normal spindle morphology and CGs function.

### RSV plays a role in mitochondrial activity

**Figure 3:**
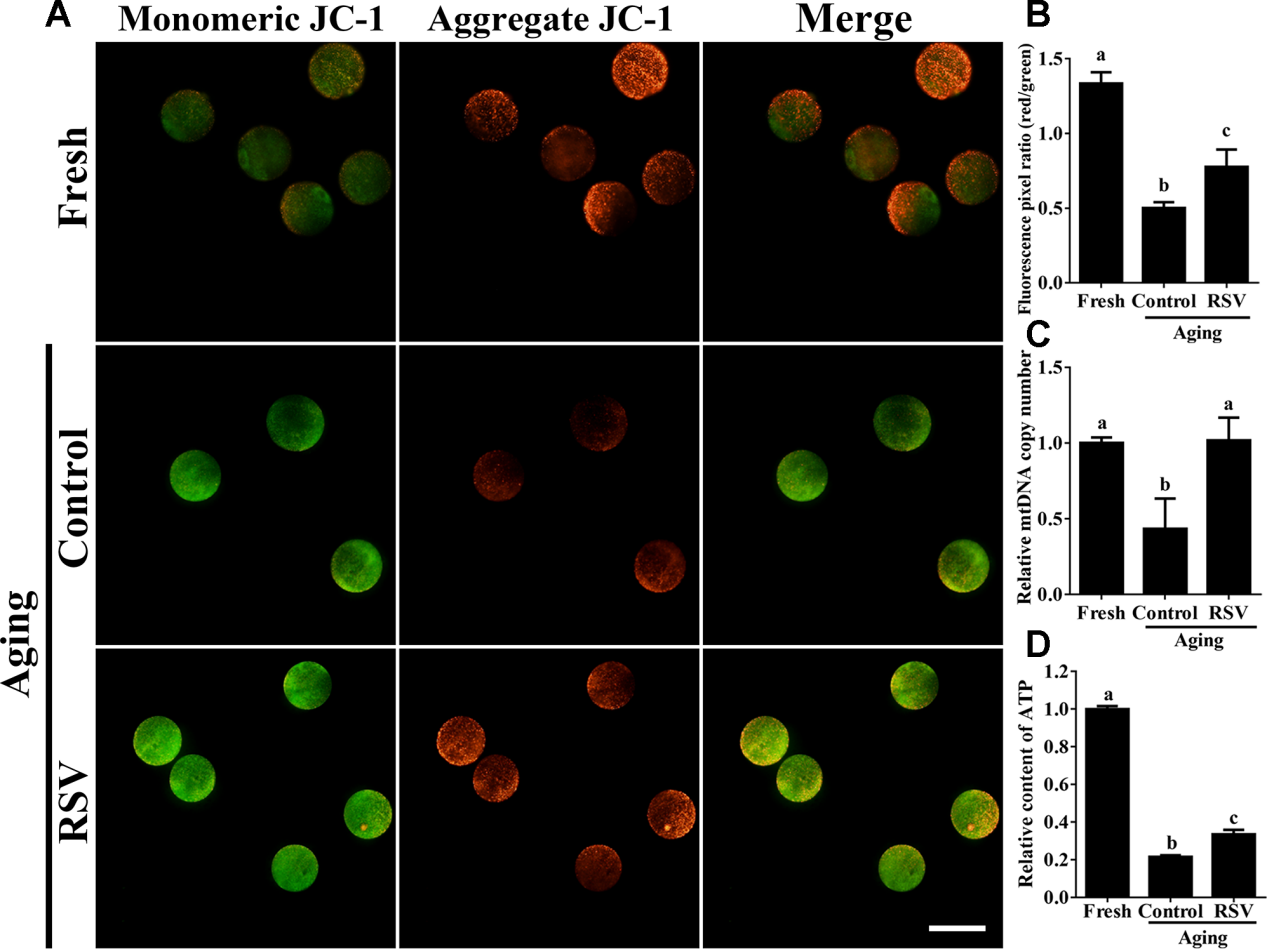
**RSV regulates mitochondrial function in aged oocytes.** (**A**) After treatment with RSV, the oocytes were stained with JC-1. Mitochondria that had high MMP were stained red while mitochondria that had low MMP were stained green. Bar = 100 μm. (**B**) Quantitative analysis of MMP in (**A**). (**C**) The effect of RSV on mtDNA copy numbers. The relative mtDNA copy numbers were detected by real time qPCR and normalized to the amount of β-globin. (**D**) The effect of RSV on ATP production. ATP level was detected as described in the material and method section. All the experiments were conducted in triplicate, and the relative expression data were normalized to embryo numbers per sample. Data are presented as means ± S.E.M of three independent experiments. Different lowercase letters represent the difference of expression levels that are significant (*P* < 0.05).

Previous results suggested that oxidative stress triggered oocyte aging while mitochondrial DNA damage led to the dysplasia [22]. We thus determined mitochondrial activity in both control and RSV-treated oocytes. JC-1 staining experiments indicated that the mitochondrial membrane potential (MMP) was significantly decreased in aged oocytes compared to that of fresh oocytes (0.50 ± 0.04 vs. 1.34 ± 0.08; *p* < 0.05); however, the decreased MMP was partially restored in RSV-treated aged oocytes (0.78 ± 0.04 vs. 0.50 ± 0.04; *p* < 0.05) (Figure 3A and 3B). To further confirm this result, we determined the mtDNA copy numbers and ATP production among different groups. Consistently, results indicated that the decreased mtDNA and ATP production in aged oocytes were restored (or partially restored) upon RSV administration (0.77 ± 0.05 vs. 0.49 ± 0.09; *p* < 0.05) (0.34 ± 0.02 vs. 0.22 ± 0.01; *p* < 0.05) (Figures 3C and 3D). Therefore, our results suggested that RSV played an important role in protecting mitochondrial function during oocyte aging.

### RSV promotes mitophagy during postovulatory oocyte aging

**Figure 4:**
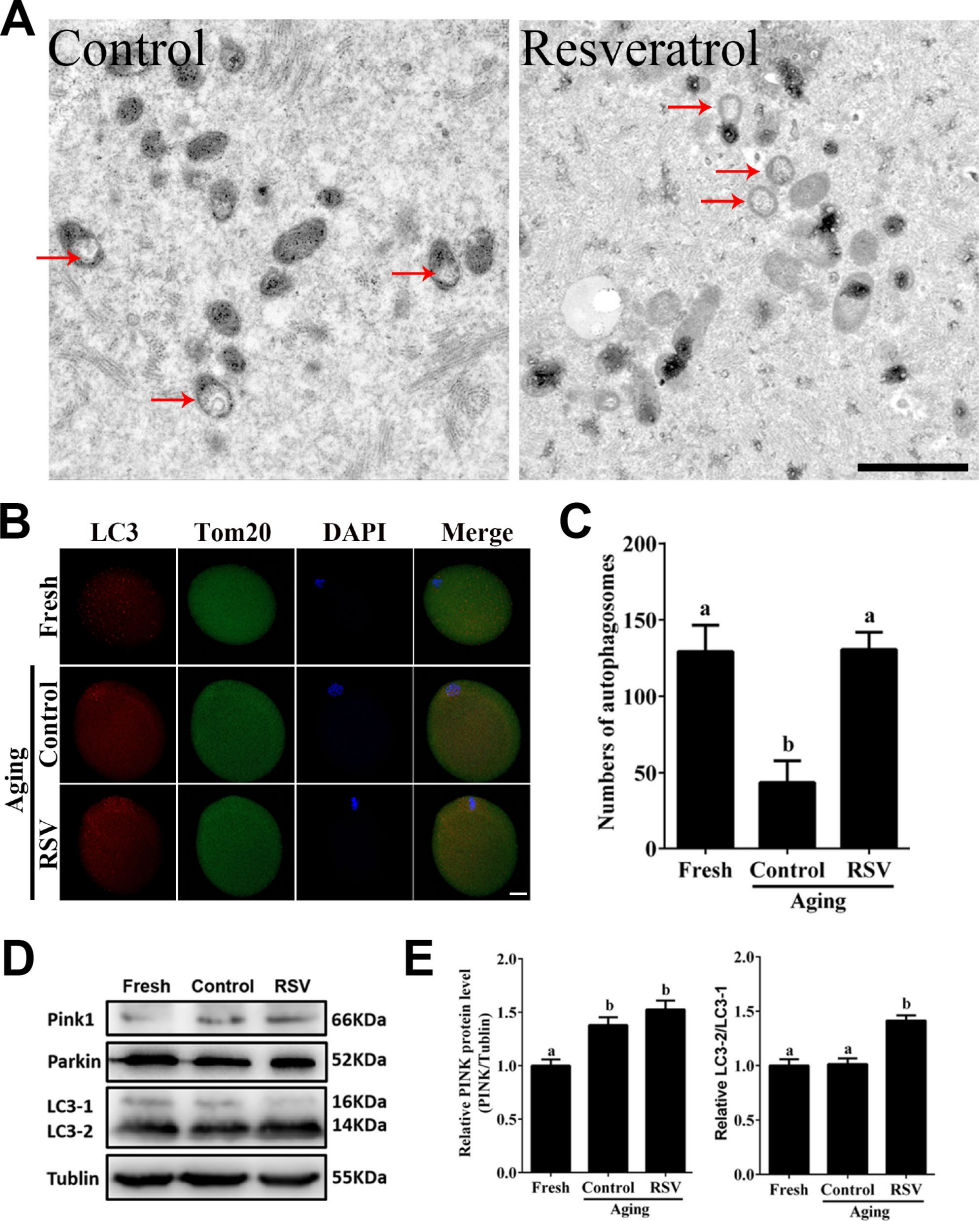
**RSV promotes mitophagy in aged oocytes.** (**A**) Electron microscopy results showed the effect of RSV on mitochondrial morphology. Bar = 1 μm. (**B**) With the treatment of RSV, the autophagy level was detected by counterstaining with LC3 (red), Tom20 (green) and DAPI (blue). Bar = 50 μm. (**C**) Quantitative analysis of autophagosome numbers in (**B**). (**D**) After RSV treatment, the LC3 (including LC3-1 and LC3-2), PINK and Parkin protein expressions were measured by western blot. α-Tubulin was used as a loading control. (**E**) Quantitative analysis of protein level of PINK1 in (**D**) and the relative LC3-2/1 level was calculated. Data are presented as means ± S.E.M of three independent experiments. Different lowercase letters represent the difference of expression levels that are significant (*P* < 0.05).

Mitophagy is critical for maintaining normal mitochondria quality and quantity [17]. To investigate the involvement of mitophagy in RSV-treated aged oocytes, we next analyzed mitochondrial morphology and the occurrence of mitophagy in both the control and RSV group. Transmission electron microscopy (TEM) showed that in aged oocytes, mitochondria lost their cristae and formed large vacuoles (Figure 4A). In RSV-treated oocytes, however, mitochondria were sequestered into an autophagosome, reflecting an upregulated mitophagy level upon RSV treatment. In addition, we detected the autophagy marker LC3 and mitochondrial marker Tom20 by immunofluorescent staining, respectively. As shown in Figures 4B and 4C, the RSV-treated oocytes displayed increased LC3 punctas representing the formation of autophagosomes when compared to control oocytes (130.6 ± 11.3, n=25 vs. 43.6 ± 14.2, n=30; *p* < 0.05). Consistently, western blot assays showed that RSV promoted mitophagy levels by activating the PINK/Parkin signaling (Figure 4D), as indicated with increased PINK expression and LC3-1 to LC3-2 conversion (Figure 4E). Together, our data clearly demonstrated that RSV could upregulate mitophagy levels during oocyte aging.

### RSV regulates oocyte aging through activating mitophagy

**Figure 5:**
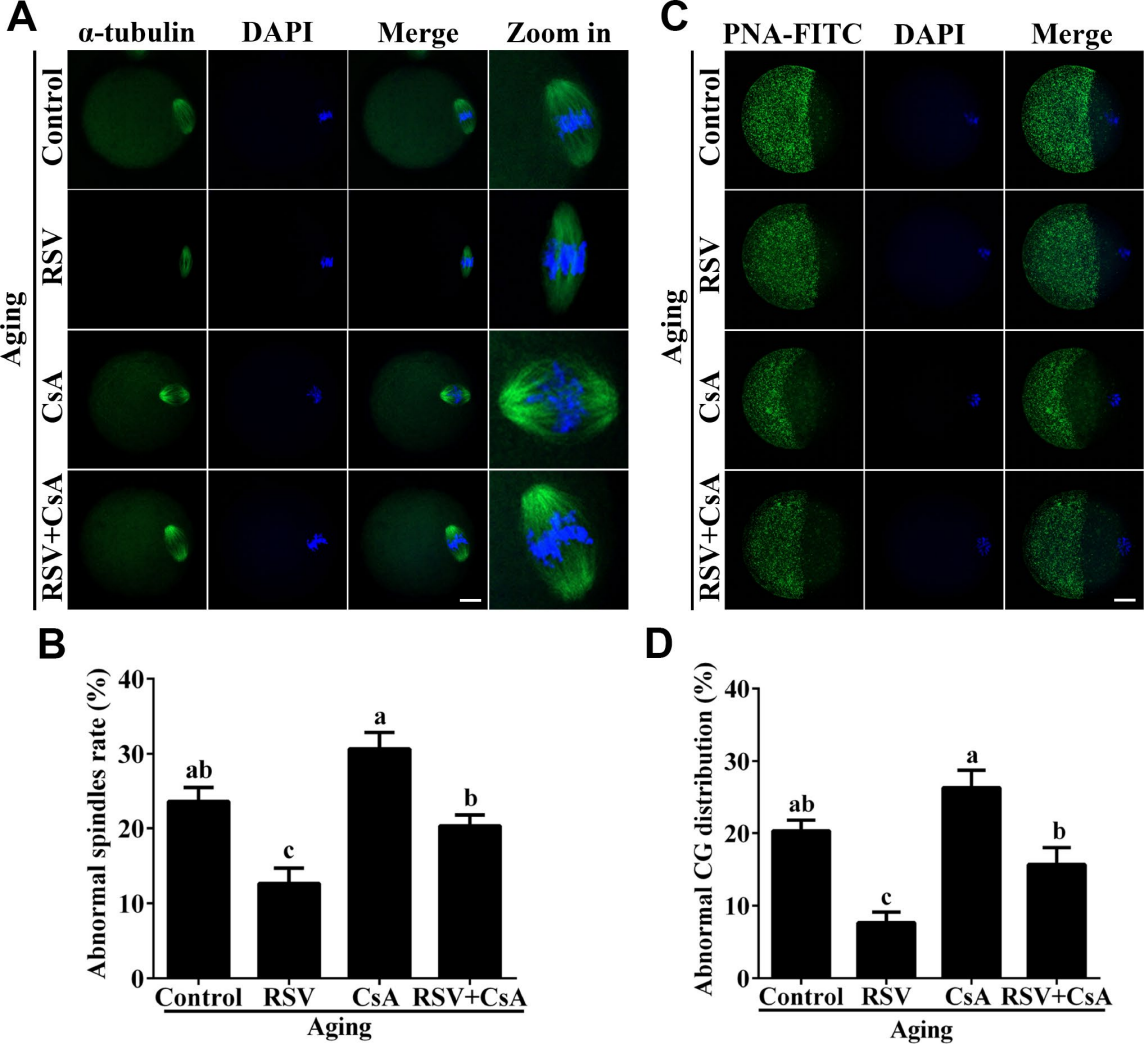
**RSV-induced mitophagy regulates spindle morphology and CG distribution.** (**A**) The effect of RSV-induced mitophagy on spindle morphology. After mitophagy blocking by CsA, the spindles were stained by anti-α-tubulin (green), and the chromosomes were counterstained with DAPI. Bar = 50 μm. (**B**) Quantitative analysis of abnormal spindle rates in (**A**). (**C**) The effect of RSV-induced mitophagy on CG distribution. After mitophagy blocking by CsA, the CG distribution was determined by staining with anti-PNA (green), and the chromosomes were counterstained with DAPI. Bar = 50 μm. (**D**) Quantitative analysis of abnormal CG distribution rate in (**C**). All the experiments were conducted in triplicates. Data are presented as means ± S.E.M of three independent experiments. Different lowercase letters represent the difference of expression levels that are significant (P < 0.05).

**Figure 6:**
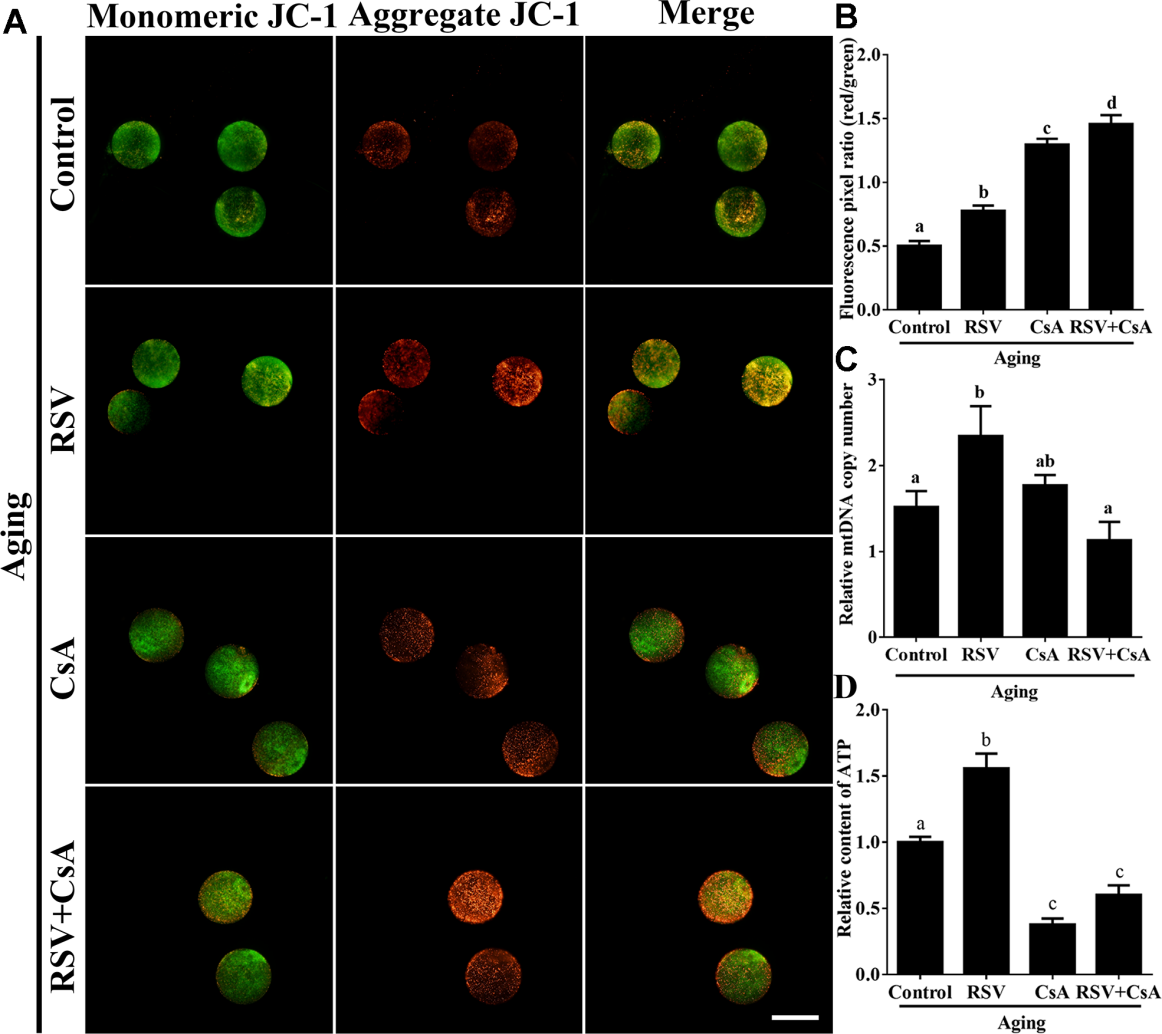
**The effect of RSV-induced mitophagy on mitochondrial function.** (**A**) After treatment with CsA, the MMP was detected by JC-1 staining. Mitochondria that had high MMP were stained red while mitochondria that had low MMP were stained green. Bar = 100 μm. (**B**) Quantitative analysis of MMP in (**A**). (**C**) The effect of RSV induced mitophagy on mtDNA copy numbers. The relative mtDNA copy numbers were detected by real time qPCR and normalized to the amount of β-globin. (**D**) The effect of RSV-induced mitophagy on ATP production. ATP level was detected as described in the material and method section. All the experiments were conducted in triplicate, and the relative expression data were normalized to embryo numbers per sample. Data are presented as means ± S.E.M of three independent experiments. Different lowercase letters represent the difference of expression levels that are significant (*P* < 0.05).

**Figure 7:**
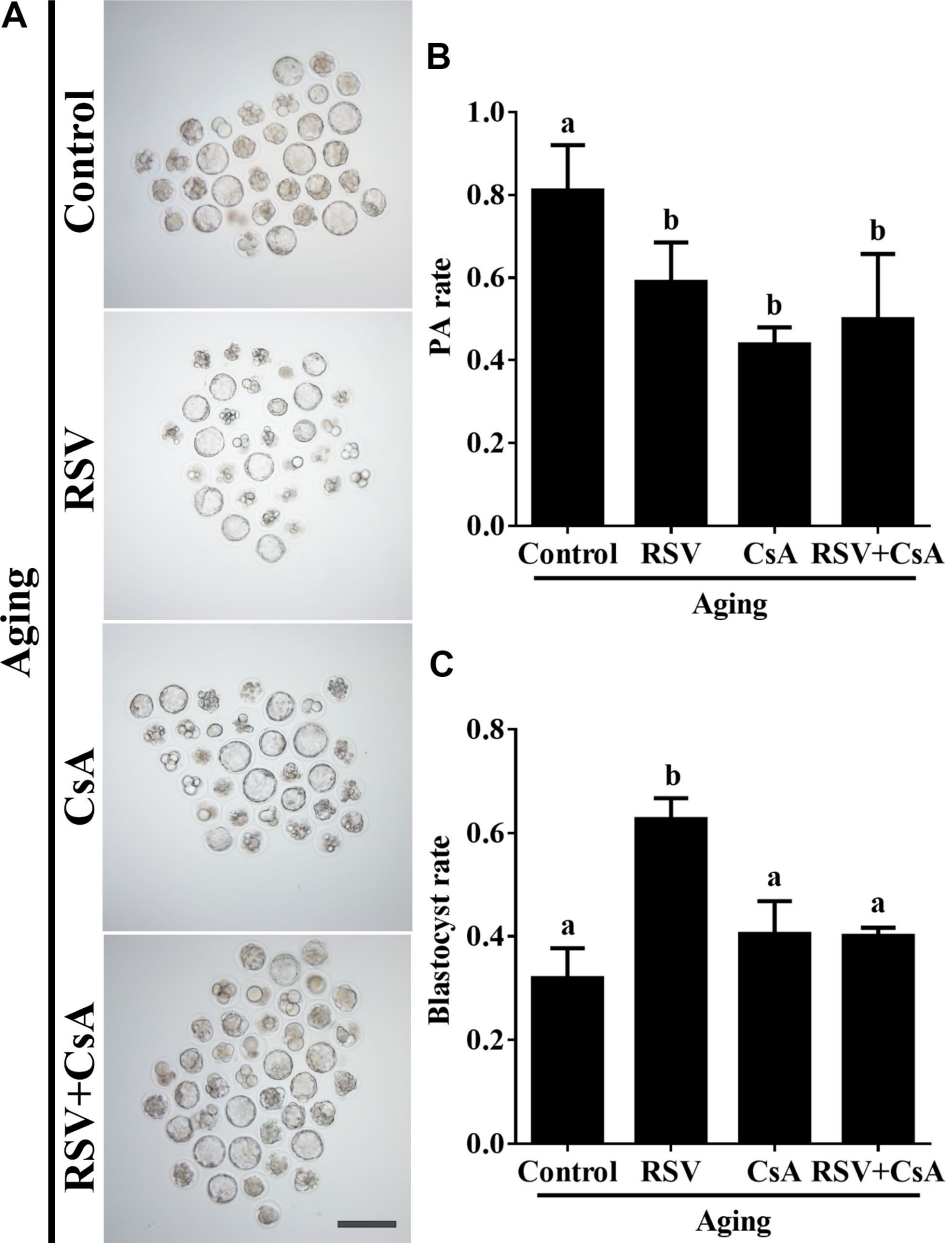
**The effect of RSV-induced mitophagy on developmental potential of aged oocytes.** (**A**) After treatment with CsA, the blastocyst formation rate was detected. Bar = 200 μm. (**B**) Statistical results of the parthenogenetic-activation rate after RSV induced mitophagy blocked by CsA. (**C**) Quantitative analysis of blastocyst formation rates in (**A**). At least three independent experiments and more than 20 embryos were examined in each experimental group. Different lowercase letters represent the difference of expression levels that are significant (*P* < 0.05).

**Figure 8:**
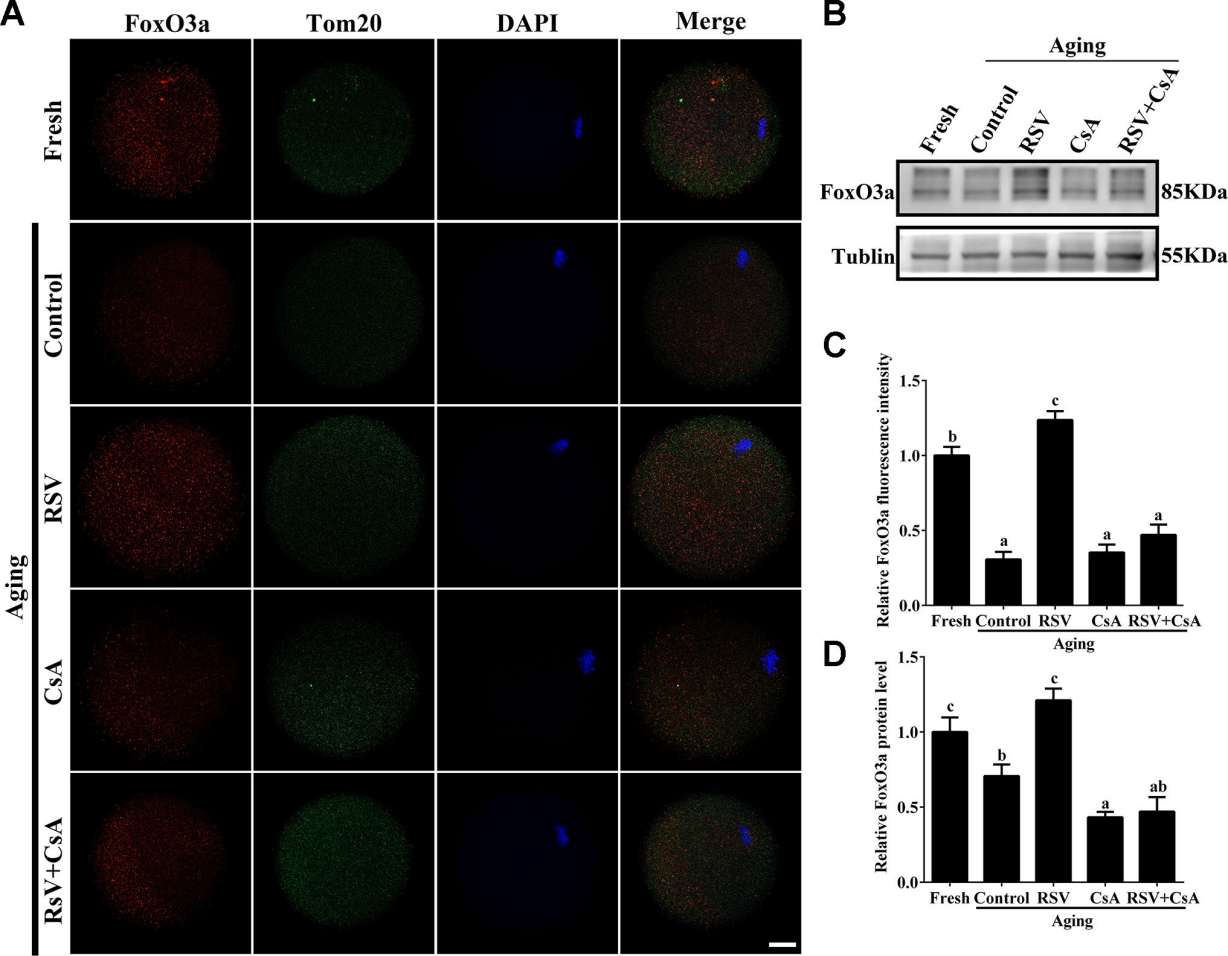
**The involvement of FoxO3a in RSV-mediated mitophagy during oocyte aging.** (**A**) With the treatment of RSV and CsA, the FoxO3a expression was determined by immunostaining with FoxO3a (red), Tom20 (green) and DAPI (blue). Bar = 50 μm. (**B**) After the indicated treatment of RSV and CsA, the FoxO3a protein expressions were measured by western blot. α-Tubulin was used as a loading control. (**C**) Quantitative analysis of FoxO3a fluorescence intensity in (**A**). (**D**) Quantitative analysis of FoxO3a protein expression in (**B**). All experiments were conducted in triplicate. Data are presented as means ± S.E.M of three independent experiments. Different lowercase letters represent the difference of expression levels that are significant (*P* < 0.05).

To further demonstrate the important role of mitophagy in oocyte aging, Cyclosporin A (CsA), a well-defined mitophagy inhibitor was used to block the mitochondrial permeability transition, inhibit mitochondrial depolarization and autophagosomal proliferation [23]. As expected, immunofluorescence staining indicated that the LC3 punctas were significantly decreased after CsA treatment (Supplementary Figure 3). We next investigated whether RSV-mediated mitophagy is correlated with oocyte aging. As shown in Figure 5, the maintenance role of RSV in spindle morphology (Abnormal rate: 20.33 ± 1.5, n = 83 vs. 12.67 ± 2.0, n = 91; *p* < 0.05) and CG distribution (Abnormal rate: 15.67% ± 2.3, n = 71 vs. 7.67% ± 1.5, n =88; *p* < 0.05) was abolished after blocking mitophagy in CsA-treated oocytes. In addition, we also found that the mitochondrial functions including MMP, mtDNA copy numbers and ATP production were significantly affected by CsA treatment (Figure 6), suggesting a protective role of RSV in maintaining mitochondrial function through activating mitophagy. Consistent results were obtained by determining the blastocyst formation of oocytes following CsA treatment (40.1% ± 1.5, n=109 vs. 59.9% ± 4.1, n=94; *p* < 0.05) (Figures 7A and 7C), further confirming that the mitophagy upregulated by RSV is an important mechanism to improve oocyte quality and developmental competence. Therefore, our results suggested that RSV regulated oocyte aging through activating mitophagy. As one of the downstream signaling molecules of RSV, FoxO3a has been reported to upregulate mitophagy by directly or indirectly targeting some autophagy related genes including *LC3* and *Bnip3* [24]. We thus examined whether FoxO3a is required for RSV-mediated mitophagy in oocyte aging. As shown in Figure 8, RSV treatment increased the expression of FoxO3a and upregulated FoxO3a expression was positively associated with RSV-induced autophagy signaling (Figure 4B and 4D). In contrast, inhibited FoxO3a expression was observed in CsA-treated aged oocytes under RSV conditions, suggesting a blockade of FoxO3a-induced mitophagy. Overall, these data suggest that FoxO3a is a critical factor involved in RSV-mediated mitophagy during postovulatory oocyte aging.

## DISCUSSION

Since the birth of the first child conceived through ART in 1978, this emerging technology has helped a great number of infertility-affected couples achieve their parenting dream. To date, a series of ART technologies, including *in vitro* maturation (IVM), *in vitro* fertilization (IVF) and intracytoplasmic sperm injection (ICSI) have been developed and improved continuously. However, oocyte aging during ART procedures is still a problem that is hard to address. In animal models, oocyte aging is associated with a series of molecular, biochemical and functional changes that may affect not only pre- and post-implantation embryo development but also later life of the offspring [5]. In this study, we investigated whether RSV, a natural potential anti-aging polyphenolic compound, has an effect on postovulatory oocyte aging *in vitro*, which might provide a theoretical basis for clinical studies in human ART.

Accumulating evidences have revealed that RSV has potential beneficial effects on reproductive functions. For postovulatory aging, these beneficial effects typically occurred through improving developmental potential and alteration of spindle morphology and CG distribution. Previous reports suggested that the rates of development to blastocysts, as well as the total cell numbers of blastocysts in mice were increased after treatment with RSV [10]. Consistently, our results showed that 10μM RSV treatment significantly promotes blastocyst formation and blastocyst cell numbers (Figure 1A). Spindle morphology and CG distribution were two critical criterions to evaluate oocyte quality, since an intact spindle is necessary for accurate chromosome segregation while uniform distribution of CGs is necessary to prevent polyspermy. Our previous report indicated that aged oocytes exhibited elongated and/or smaller spindle morphologies and a partial CG release [1]. Here, we showed that RSV prevented these abnormal changes and restored spindle morphology and displayed typical CG distribution compared to those seen in fresh oocytes (Figure 2). These data are also in agreement with an earlier report suggesting that RSV protects porcine oocytes against *in vitro* aging through decreasing the frequency of spindle defects and chromosome misalignments [25], further suggesting a protective role for normal embryonic development.

Mitochondria are the principal energy producers in oocytes, using the oxidative phosphorylation pathway to convert nutrients into ATP for all energy requiring cellular activities [26]. During oocyte maturation and embryonic development, the pattern of mitochondrial distribution is a highly dynamic process both in numbers and locations [27]. Inadequate redistribution of mitochondria throughout the ooplasm has been strongly linked to low developmental potential [28]. Importantly, mitochondria are inherited maternally and independently of the nuclear genome, therefore high quality of mitochondria is required to ensure the survival and viability of the offspring. Our current findings showed that RSV treatment restored mitochondrial functions including MMP, mtDNA and ATP production during oocyte aging (Figure 3), suggesting that RSV improves oocyte quality and embryonic development through regulating mitochondrial function.

Mitophagy is a critical mitochondrial quality control mechanism that selectively eliminates damaged or excessive mitochondria through autophagosome degradation [29]. Although significant progress has been made towards understanding the precise mechanisms of mitophagy to ensure oocyte quality, the role of RSV in promoting mitophagy against oocyte aging remains unknown. In the present study, we suggested that the LC3 punctas, representing autophagosome formations, were upregulated following RSV treatment (Figure 4B). In addition, we also found that the improved spindle morphology and CG distribution state following RSV treatment was abolished after mitophagy inhibition (Figure 5), which was consistent with decreased mitochondrial function and developmental potential (Figure 6 and 7). Therefore, these data for the first time demonstrate that RSV delays postovulatory oocyte aging through regulating mitophagy.

FoxO3a, which plays an essential role in human longevity, is also involved in oocyte aging [30]. It was indicated that FoxO3a is a mitochondrial protein and forms a physical interaction with SIRT3 in mitochondria, which further promotes mitophagy against cellular oxidation stress [31]. In this study, we found that FoxO3a expression was upregulated and showed a similar expression pattern of LC3 in aged oocytes upon RSV administration (Figure 8; Figure 4B). In addition, blocking mitophagy by CsA decreased the expression of FoxO3, further suggesting that FoxO3a was required for RSV-mediated mitophagy in oocyte aging. Collectively, our work might provide evidence for the involvement of FoxO3a in RSV-mediated mitophagy regulation during oocyte aging.

In summary, our results revealed that RSV could delay oocyte aging and improve developmental competence through activating mitophagy, which serves as an important mechanism to regulate mitochondrial function. These findings not only deeply clarify the molecular mechanism of RSV in regulating oocyte aging, but also provide some theoretical basis for clinical human ART and agricultural animal embryo engineering.

## MATERIALS AND METHODS

### Oocyte collection and culture conditions

All animal experiments were conducted with the approval of the Animal Care and Use Committee of Huazhong Agriculture University, China. Six-to-eight-weeks-old female ICR mice were maintained five per cage in a temperature-controlled (22 ± 2°C) room under a 12 h light/12 h dark schedule (lights on from 07: 00 to 19: 00) with a continuous supply of water and food. Mice were administrated intraperitoneal injections of 10 IU pregnant mare’s serum gonadotropin (PMSG) followed 48 h later by human chorionic gonadotropin (hCG, 10 IU) (both from Ningbo Hormone Product Co. Ltd., Cixi, China). The superovulated mice were killed 13 hours of hCG injection, and the oviductal ampullae were broken with a syringe to release cumulus oocyte complexes (COCs). To induce oocyte aging, the COCs were dispersed and cultured *in vitro* in Chatot-Ziomek-Bavister (CZB) medium for 8 hours. For the RSV-treated group, the concentration of RSV in CZB was 10 μM. In order to get cumulus-denuded oocytes, COCs were transferred into PBS medium containing 0.1% hyaluronidase and cumulus cells were removed by gentle pipetting.

### Chemicals and reagents

RSV (R5010), 6-DMAP (D2629) and CsA (C3662) were purchased from Sigma Chemical Co. and solubilized in DMSO. The JC-1 (5, 5’, 6, 6’-tetrachloro-1, 1’, 3, 3’ tetra ethylbenzymidazolyl carbocyanine iodide) mitochondrial membrane potential (ΔΨm, MMP) kit and ATP assay kit were purchased from Beyotime Institute of Biotechnology (Haimen, China). Basic maturation culture medium was K-modified simplex optimized medium (KSOM) (Chemicon International, Inc, Temecula, CA, USA). Phosphate buffer saline (PBS) was obtained from Life Technologies (Invitrogen, Carlsbad, CA, USA).

### Immunofluorescence staining

At least 20 embryos were fixed in 4% (W/V) paraformaldehyde, permeabilized with 1% Triton X-100 in PBS, blocked with 3% BSA for 1 h at room temperature (RT), respectively. For spindle shape detection, oocytes were stained with anti-α-tubulin-FITC antibodies (diluted 1:800; F2168, Sigma Aldrich) for 30 min. For CGs distribution detection, embryos were stained with anti-PNA-FITC antibodies (diluted 1:120; L7381, Sigma Aldrich) for 30 min. For autophagosome detection, oocytes were incubated with anti-LC3 antibodies (diluted 1:100; catalog no. 3868, Cell Signal Technology) and TOM 20 antibodies (diluted 1:100; catalog no. 12741, Cell Signal Technology) for 1 h. For FoxO3a detection, oocytes were incubated with anti-FoxO3a antibodies (diluted 1:100; catalog no. 12829, Cell Signal Technology) and TOM 20 antibodies (diluted 1:100; catalog no. 12741, Cell Signal Technology) for 1 h at RT and incubated with anti-rabbit IgG conjugated with Alexa Fluor 549 (red) and anti-mouse IgG conjugated with Alexa Fluor 488 (green) for another 1 h in the dark. The coverslips were finally mounted on glass slides with 10 mg/ml DAPI (Beyotime Institute of Biotechnology, Haimen, China). Fluorescent images were taken using a laser-scanning confocal microscope (Carl Zeiss, Göttingen, Germany).

### Parthenogenetic activation

Only oocytes with first polar bodies were treated for parthenogenetic activation. The denuded oocytes were first treated with 5% (v/v) ethanol in M2 medium for 5 min at RT, then washed three times and cultured in CZB containing 2 mM 6-DMAP for 6 h. At the end of culture, the activation of oocytes was observed under a microscope. Only those eggs with one pronucleus or two pronuclei, or two cells each having a nucleus, were considered activated. Oocytes for controls were cultured for 8 h in CZB containing no 6-DMAP without prior ethanol treatment. Controls were set for each experiment and data were used only when no control oocytes were activated in the experiments.

### JC-1 staining

The mitochondrial membrane potential assay kit with JC-1 (Beyotime Institute of Biotechnology, Haimen, China) was used to evaluate the mitochondrial membrane potential. Oocytes were exposed to 10 μM JC-1 in 100 μl working solution at 37.5°C in 5% CO_2_ for 30 min, after which they were washed with CZB to remove surface fluorescence, and then mounted on glass slides for microscopy. Laser excitation was set at 488 nm for green and 525 nm for red fluorescence, respectively. The fluorescence intensity in each oocyte was measured under a fluorescence microscope (Olympus, Tokyo, Japan) with the same scan settings for each sample. The normal fluorescence pixel intensities of each oocyte were analyzed using ImageJ software (NIH, Bethesda, MD, USA). The ratio of red to green fluorescence pixels was used to analyze mitochondrial membrane potential.

### Determination of ATP content

ATP content of oocytes was determined with the ATP Testing Assay Kit (Beyotime Institute of Biotechnology, Haimen, China) according to the manufacturer’s instructions. Briefly, oocytes were lysed in ATP lysis buffer (from the kit) and centrifuged at 12,000 rcf for 10 min. Supernatants were mixed with testing buffer, and ATP concentrations were measured on a luminescence detector. The experiments were conducted in triplicate, and the results were normalized to cell numbers per sample.

### Transmission electron microscopy

Fixation was performed in 2.5% glutaraldehyde in an Eppendorf vial for 2 days at 4°C. Oocytes were then treated with 1% agar for 40 min and dehydrated in ascending series of ethanol. After that oocytes were immersed in propylene oxide for solvent substitution, embedded in Epon 812 (Agar Scientific, Stansted, UK) and sectioned. Ultrathin sections (60–80 nm) were cut with a diamond knife, mounted on copper grids and contrasted with saturated uranyl acetate followed by lead citrate. They were examined and photographed using a FEI Tecnai 8482 Electron Microscope operating at 200 KV.

### Determination of mtDNA copy numbers

Total DNA was isolated from 30 mouse oocytes by using lysate buffer (50 mM Tris-HCl, 1 mM EDTA, 0.5% Tween-20, 100 mg/mL protease K) and incubated in 55°C for 2 h. The lysate was then heated to 90°C for 30 minutes to fully lyse, after which the samples were directly used in Real-time qPCR with the mtDNA-specifc primers (Forward: 5’-TACCTCACCATCTCT TGCTA-3’; Reverse: 5’-CCACATAGACGAGTTGAT TC-3’). The data for the mtDNA were normalized to the amount of β-globin (F: 5’-CTCCTGGGCAACGTGAT AGT-3’; R: 5’-GGTTCAGAGGAA AAAGGGCTC CTCCT-3’). The Real-time qPCR was performed with SYBR Premix Ex Taq (Takara Bio, Tokyo, Japan) in a reaction volume of 10 μl and the ABI QuantStudio 5 system (Applied Biosystems, Foster City, CA, USA).

### Western blot

After the indicated treatments, protein samples were harvested in 1 × SDS loading buffer and separated by SDS-PAGE and electrophoretically transferred to polyvinylidene fluoride membranes (Millipore, Billerica, MA, USA). Incubation followed with anti-LC3 (catalog no. L8918, Sigma-Aldrich), anti-PINK1 (catalog no. ab23707, abcam), anti-Parkin (catalog no. 2132, Cell Signal Technology) overnight at 4°C and with HRP-conjugated anti-rabbit secondary antibody (catalog no. 7074, Cell Signal Technology) or anti-mouse secondary antibody (catalog no. 7076, Cell Signal Technology) for 2 h at 25°C. After washing, the membrane was processed using SuperSignal West Pico chemiluminescent substrate (Pierce Chemical). As an internal control, α-tubulin was detected using an anti-tubulin antibody (catalog no. T5168, Sigma).

### Statistics

Results were expressed as the means ± S.E.M from at least three independent experiments. The statistical analysis was performed by t-test and one-way analysis of variance using SPSS software version 18.0 (SPSS, Chicago, IL, USA). *P* < 0.05 was considered to indicate statistical significance.

## Supplementary Material

Supplementary Figures
