# Supplementary material for: Resveratrol delays postovulatory aging of mouse oocytes through activating mitophagy
Source: Aging (Albany NY). 2019 Dec 13;11(23):11504–19. doi: 10.18632/aging.102551 (PMC6932885; doi:10.18632/aging.102551)
Supplement: Supplementary Figures [file aging-11-102551-s001..pdf]

SUPPLEMENTARY FIGURES

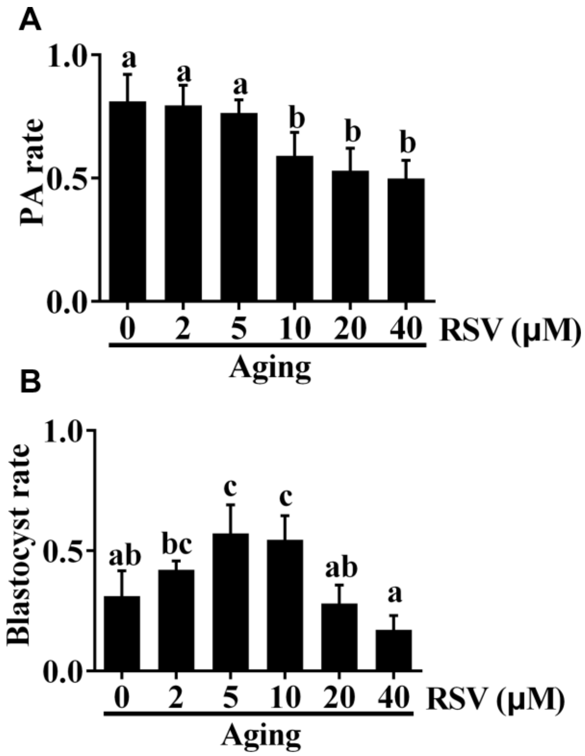

Supplementary Figure 1. The effect of different concentrations of RSV on parthenogenetic-activation rate and blastocyst formation rate. (**A**) Parthenogenetic-activation rate of oocytes under different concentrations of RSV. Oocytes were cultured in vitro for 8h with different concentrations of RSV. Activation rates of oocytes in different groups were calculated under a microscope. (**B**) The activated eggs obtained from (**A**) were cultured for additional 84 h, the blastocyst rates were calculated under a microscope. Data are presented as means ± SEM of three experiments. Different lowercase letters represent the difference of expression levels that are significant (*P* < 0.05).

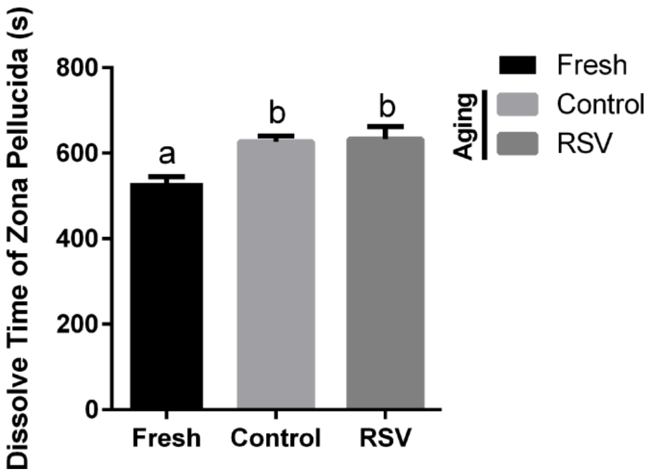

Supplementary Figure 2. Changes in chymotrypsin digestion time of ZP (T50 is the time at which 50% of the ZPs per group were completely digested) of oocytes under RSV condition. After in vitro culture for 8h with different concentrations of RSV, oocytes were transferred into CZB medium containing chymotrypsin. Digestion time was calculated under a microscope. Different lowercase letters represent the difference of expression levels that are significant (*P* < 0.05).

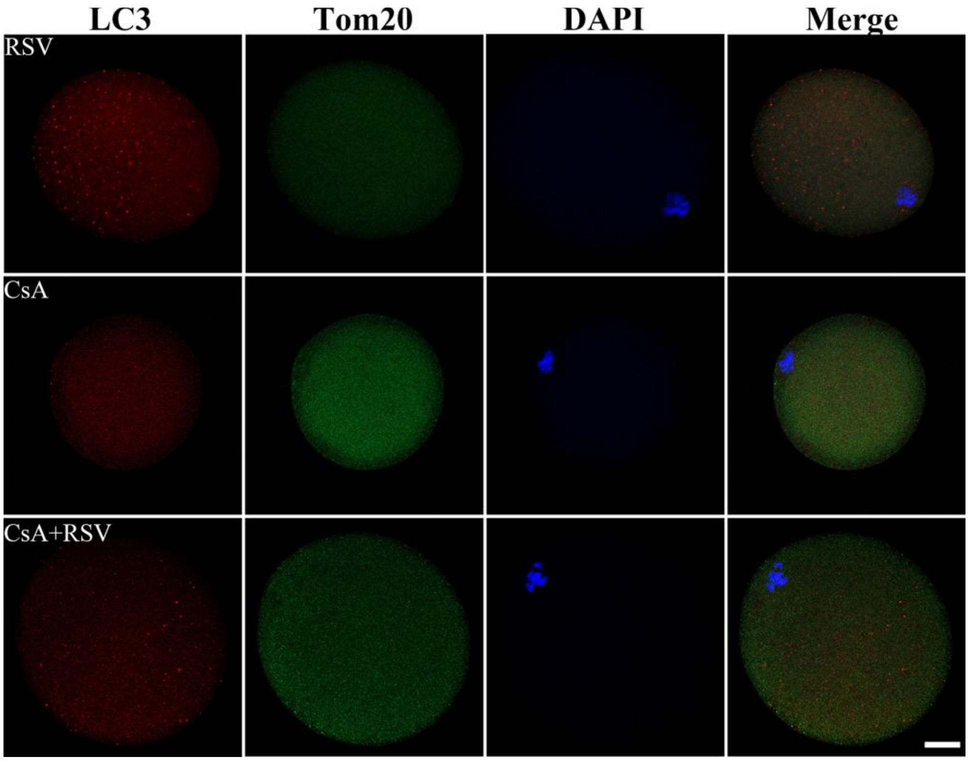

Supplementary Figure 3. CsA inhibited LC3 expression in aged oocytes. After mitophagy blocked by CsA, aged oocytes under RSV administration were immunostained by LC3 (red) and Tom20 (green). At least three independent experiments and more than 20 embryos were examined in each experimental group.
